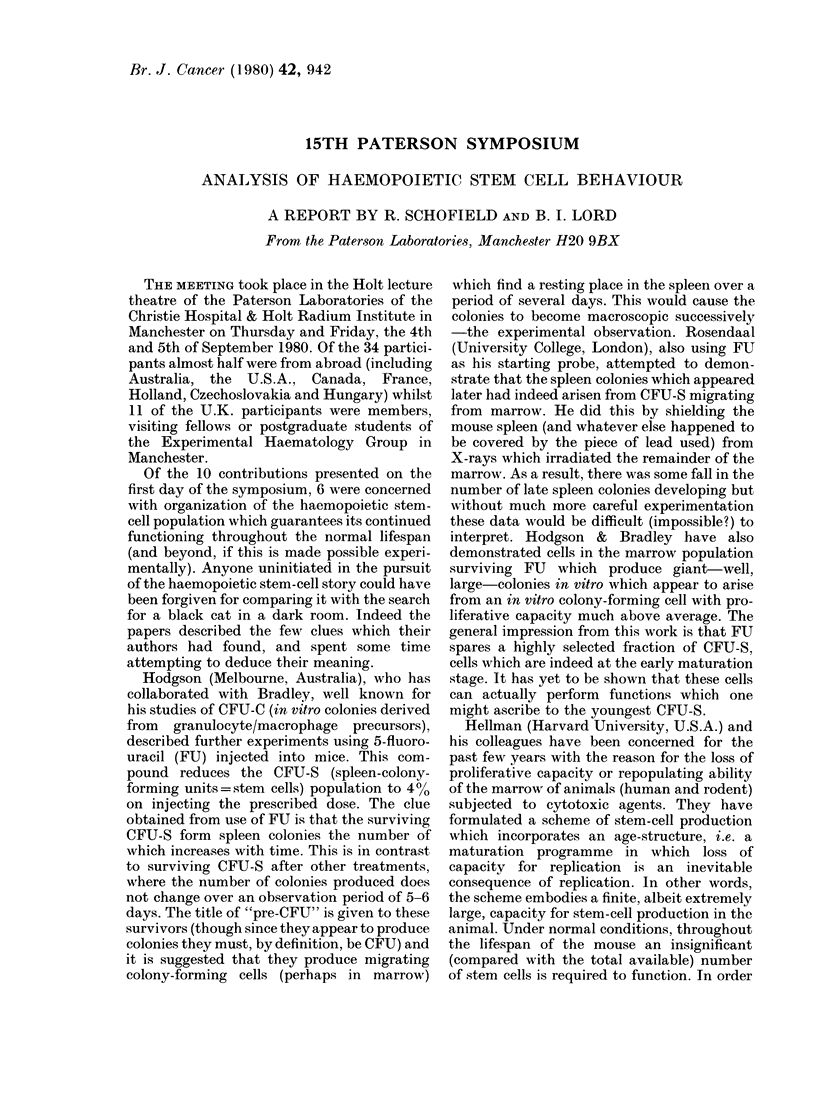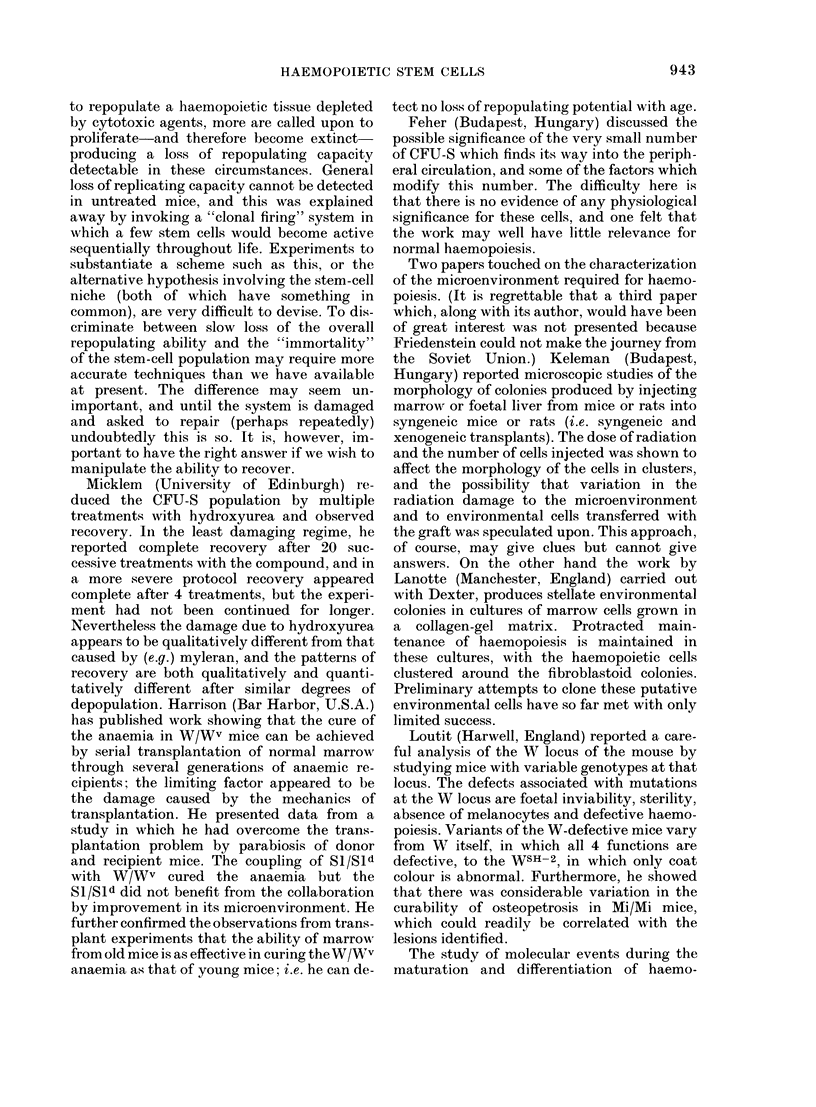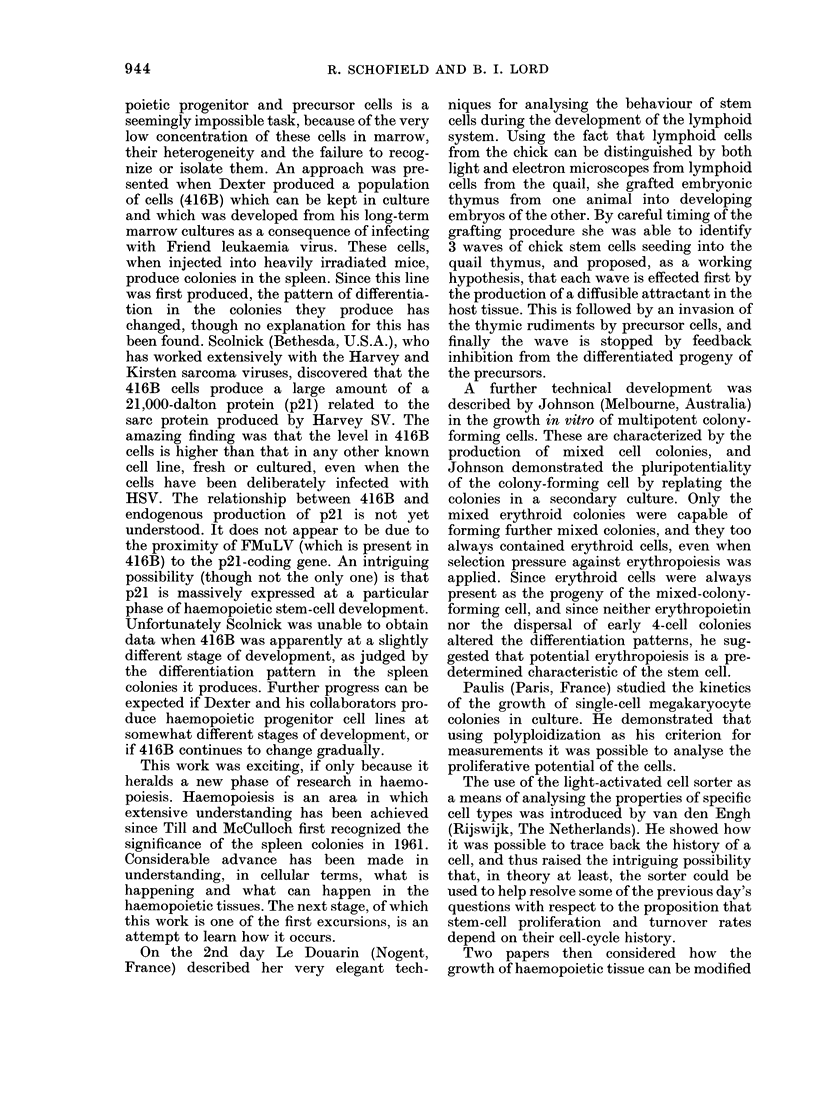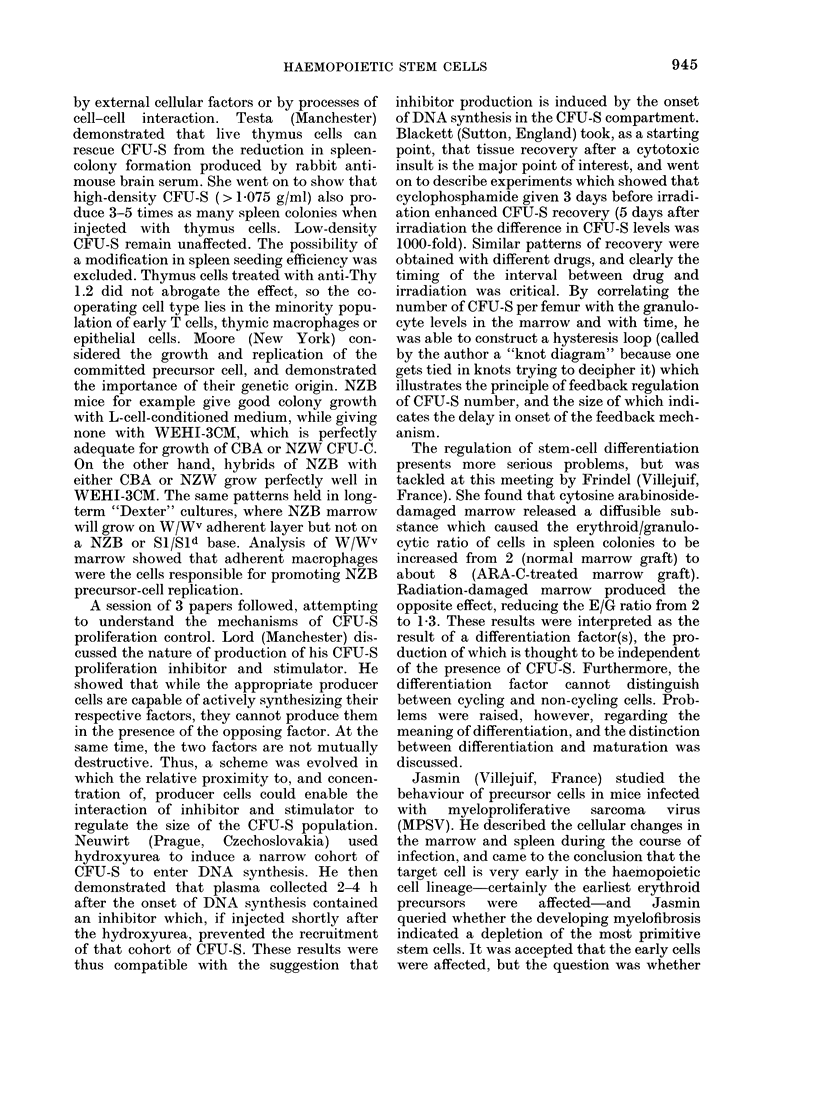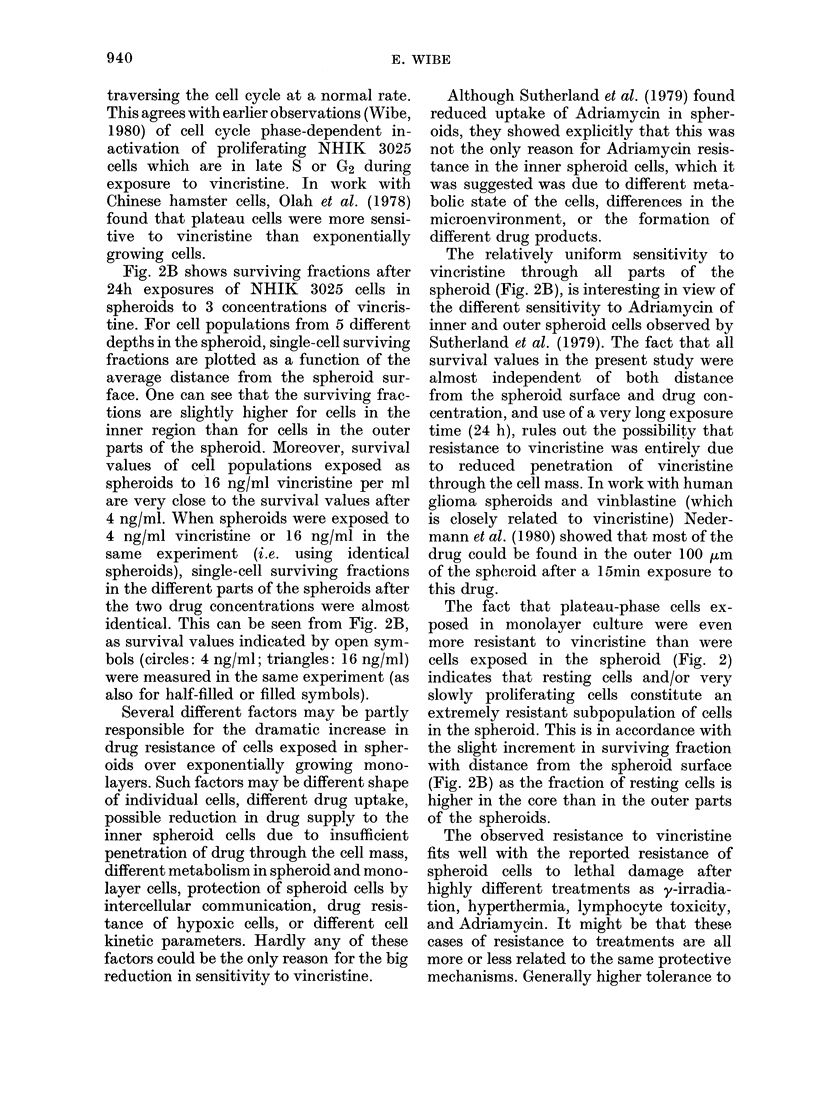# Proceedings of the 15th Paterson Symposium Analysis of haemopoietic stem cell behaviour

**Published:** 1980-12

**Authors:** 


*Br. J. Cancer* (1980) **42**, 942

### 15TH PATERSON SYMPOSIUM

### ANALYSIS OF HAEMOPOIETIC STEM CELL BEHAVIOUR

A REPORT BY R. SCHOFIELD AND B. I. LORD

*From the Paterson Laboratories, Manchester H20 9BX*

THE MEETING took place in the Holt lecture theatre of the Paterson Laboratories of the Christie Hospital & Holt Radium Institute in Manchester on Thursday and Friday, the 4th and 5th of September 1980. Of the 34 participants almost half were from abroad (including Australia, the U.S.A., Canada, France, Holland, Czechoslovakia and Hungary) whilst 11 of the U.K. participants were members, visiting fellows or postgraduate students of the Experimental Haematology Group in Manchester.

Of the 10 contributions presented on the first day of the symposium, 6 were concerned with organization of the haemopoietic stem-cell population which guarantees its continued functioning throughout the normal lifespan (and beyond, if this is made possible experimentally). Anyone uninitiated in the pursuit of the haemopoietic stem-cell story could have been forgiven for comparing it with the search for a black cat in a dark room. Indeed the papers described the few clues which their authors had found, and spent some time attempting to deduce their meaning.

Hodgson (Melbourne, Australia), who has collaborated with Bradley, well known for his studies of CFU-C (*in vitro* colonies derived from granulocyte/macrophage precursors), described further experiments using 5-fluorouracil (FU) injected into mice. This compound reduces the CFU-S (spleen-colony-forming units = stem cells) population to 4% on injecting the prescribed dose. The clue obtained from use of FU is that the surviving CFU-S form spleen colonies the number of which increases with time. This is in contrast to surviving CFU-S after other treatments, where the number of colonies produced does not change over an observation period of 5–6 days. The title of "pre-CFU" is given to these survivors (though since they appear to produce colonies they must, by definition, be CFU) and it is suggested that they produce migrating colony-forming cells (perhaps in marrow) which find a resting place in the spleen over a period of several days. This would cause the colonies to become macroscopic successively—the experimental observation. Rosendaal (University College, London), also using FU as his starting probe, attempted to demonstrate that the spleen colonies which appeared later had indeed arisen from CFU-S migrating from marrow. He did this by shielding the mouse spleen (and whatever else happened to be covered by the piece of lead used) from X-rays which irradiated the remainder of the marrow. As a result, there was some fall in the number of late spleen colonies developing but without much more careful experimentation these data would be difficult (impossible?) to interpret. Hodgson & Bradley have also demonstrated cells in the marrow population surviving FU which produce giant—well, large—colonies *in vitro* which appear to arise from an *in vitro* colony-forming cell with proliferative capacity much above average. The general impression from this work is that FU spares a highly selected fraction of CFU-S, cells which are indeed at the early maturation stage. It has yet to be shown that these cells can actually perform functions which one might ascribe to the youngest CFU-S.

Hellman (Harvard University, U.S.A.) and his colleagues have been concerned for the past few years with the reason for the loss of proliferative capacity or repopulating ability of the marrow of animals (human and rodent) subjected to cytotoxic agents. They have formulated a scheme of stem-cell production which incorporates an age-structure, *i.e.* a maturation programme in which loss of capacity for replication is an inevitable consequence of replication. In other words, the scheme embodies a finite, albeit extremely large, capacity for stem-cell production in the animal. Under normal conditions, throughout the lifespan of the mouse an insignificant (compared with the total available) number of stem cells is required to function. In order to repopulate a haemopoietic tissue depleted by cytotoxic agents, more are called upon to proliferate—and therefore become extinct—producing a loss of repopulating capacity detectable in these circumstances. General loss of replicating capacity cannot be detected in untreated mice, and this was explained away by invoking a "clonal firing" system in which a few stem cells would become active sequentially throughout life. Experiments to substantiate a scheme such as this, or the alternative hypothesis involving the stem-cell niche (both of which have something in common), are very difficult to devise. To discriminate between slow loss of the overall repopulating ability and the "immortality" of the stem-cell population may require more accurate techniques than we have available at present. The difference may seem unimportant, and until the system is damaged and asked to repair (perhaps repeatedly) undoubtedly this is so. It is, however, important to have the right answer if we wish to manipulate the ability to recover.

Micklem (University of Edinburgh) reduced the CFU-S population by multiple treatments with hydroxyurea and observed recovery. In the least damaging regime, he reported complete recovery after 20 successive treatments with the compound, and in a more severe protocol recovery appeared complete after 4 treatments, but the experiment had not been continued for longer. Nevertheless the damage due to hydroxyurea appears to be qualitatively different from that caused by (*e.g.*) myleran, and the patterns of recovery are both qualitatively and quantitatively different after similar degrees of depopulation. Harrison (Bar Harbor, U.S.A.) has published work showing that the cure of the anaemia in $W/W^v$ mice can be achieved by serial transplantation of normal marrow through several generations of anaemic recipients; the limiting factor appeared to be the damage caused by the mechanics of transplantation. He presented data from a study in which he had overcome the transplantation problem by parabiosis of donor and recipient mice. The coupling of $Sl/Sl^d$ with $W/W^v$ cured the anaemia but the $Sl/Sl^d$ did not benefit from the collaboration by improvement in its microenvironment. He further confirmed the observations from transplant experiments that the ability of marrow from old mice is as effective in curing the $W/W^v$ anaemia as that of young mice; *i.e.* he can detect no loss of repopulating potential with age.

Feher (Budapest, Hungary) discussed the possible significance of the very small number of CFU-S which finds its way into the peripheral circulation, and some of the factors which modify this number. The difficulty here is that there is no evidence of any physiological significance for these cells, and one felt that the work may well have little relevance for normal haemopoiesis.

Two papers touched on the characterization of the microenvironment required for haemopoiesis. (It is regrettable that a third paper which, along with its author, would have been of great interest was not presented because Friedenstein could not make the journey from the Soviet Union.) Keleman (Budapest, Hungary) reported microscopic studies of the morphology of colonies produced by injecting marrow or foetal liver from mice or rats into syngeneic mice or rats (*i.e.* syngeneic and xenogeneic transplants). The dose of radiation and the number of cells injected was shown to affect the morphology of the cells in clusters, and the possibility that variation in the radiation damage to the microenvironment and to environmental cells transferred with the graft was speculated upon. This approach, of course, may give clues but cannot give answers. On the other hand the work by Lanotte (Manchester, England) carried out with Dexter, produces stellate environmental colonies in cultures of marrow cells grown in a collagen-gel matrix. Protracted maintenance of haemopoiesis is maintained in these cultures, with the haemopoietic cells clustered around the fibroblastoid colonies. Preliminary attempts to clone these putative environmental cells have so far met with only limited success.

Loutit (Harwell, England) reported a careful analysis of the W locus of the mouse by studying mice with variable genotypes at that locus. The defects associated with mutations at the W locus are foetal inviability, sterility, absence of melanocytes and defective haemopoiesis. Variants of the W-defective mice vary from W itself, in which all 4 functions are defective, to the $W^{SH-2}$, in which only coat colour is abnormal. Furthermore, he showed that there was considerable variation in the curability of osteopetrosis in Mi/Mi mice, which could readily be correlated with the lesions identified.

The study of molecular events during the maturation and differentiation of haemopoietic progenitor and precursor cells is a seemingly impossible task, because of the very low concentration of these cells in marrow, their heterogeneity and the failure to recognize or isolate them. An approach was presented when Dexter produced a population of cells (416B) which can be kept in culture and which was developed from his long-term marrow cultures as a consequence of infecting with Friend leukaemia virus. These cells, when injected into heavily irradiated mice, produce colonies in the spleen. Since this line was first produced, the pattern of differentiation in the colonies they produce has changed, though no explanation for this has been found. Scolnick (Bethesda, U.S.A.), who has worked extensively with the Harvey and Kirsten sarcoma viruses, discovered that the 416B cells produce a large amount of a 21,000-dalton protein (p21) related to the sarc protein produced by Harvey SV. The amazing finding was that the level in 416B cells is higher than that in any other known cell line, fresh or cultured, even when the cells have been deliberately infected with HSV. The relationship between 416B and endogenous production of p21 is not yet understood. It does not appear to be due to the proximity of FMuLV (which is present in 416B) to the p21-coding gene. An intriguing possibility (though not the only one) is that p21 is massively expressed at a particular phase of haemopoietic stem-cell development. Unfortunately Scolnick was unable to obtain data when 416B was apparently at a slightly different stage of development, as judged by the differentiation pattern in the spleen colonies it produces. Further progress can be expected if Dexter and his collaborators produce haemopoietic progenitor cell lines at somewhat different stages of development, or if 416B continues to change gradually.

This work was exciting, if only because it heralds a new phase of research in haemopoiesis. Haemopoiesis is an area in which extensive understanding has been achieved since Till and McCulloch first recognized the significance of the spleen colonies in 1961. Considerable advance has been made in understanding, in cellular terms, what is happening and what can happen in the haemopoietic tissues. The next stage, of which this work is one of the first excursions, is an attempt to learn how it occurs.

On the 2nd day Le Douarin (Nogent, France) described her very elegant techniques for analysing the behaviour of stem cells during the development of the lymphoid system. Using the fact that lymphoid cells from the chick can be distinguished by both light and electron microscopes from lymphoid cells from the quail, she grafted embryonic thymus from one animal into developing embryos of the other. By careful timing of the grafting procedure she was able to identify 3 waves of chick stem cells seeding into the quail thymus, and proposed, as a working hypothesis, that each wave is effected first by the production of a diffusible attractant in the host tissue. This is followed by an invasion of the thymic rudiments by precursor cells, and finally the wave is stopped by feedback inhibition from the differentiated progeny of the precursors.

A further technical development was described by Johnson (Melbourne, Australia) in the growth *in vitro* of multipotent colony-forming cells. These are characterized by the production of mixed cell colonies, and Johnson demonstrated the pluripotentiality of the colony-forming cell by replating the colonies in a secondary culture. Only the mixed erythroid colonies were capable of forming further mixed colonies, and they too always contained erythroid cells, even when selection pressure against erythropoiesis was applied. Since erythroid cells were always present as the progeny of the mixed-colony-forming cell, and since neither erythropoietin nor the dispersal of early 4-cell colonies altered the differentiation patterns, he suggested that potential erythropoiesis is a predetermined characteristic of the stem cell.

Paulis (Paris, France) studied the kinetics of the growth of single-cell megakaryocyte colonies in culture. He demonstrated that using polyploidization as his criterion for measurements it was possible to analyse the proliferative potential of the cells.

The use of the light-activated cell sorter as a means of analysing the properties of specific cell types was introduced by van den Engh (Rijswijk, The Netherlands). He showed how it was possible to trace back the history of a cell, and thus raised the intriguing possibility that, in theory at least, the sorter could be used to help resolve some of the previous day's questions with respect to the proposition that stem-cell proliferation and turnover rates depend on their cell-cycle history.

Two papers then considered how the growth of haemopoietic tissue can be modified by external cellular factors or by processes of cell–cell interaction. Testa (Manchester) demonstrated that live thymus cells can rescue CFU-S from the reduction in spleen-colony formation produced by rabbit anti-mouse brain serum. She went on to show that high-density CFU-S ( > 1·075 g/ml) also produce 3–5 times as many spleen colonies when injected with thymus cells. Low-density CFU-S remain unaffected. The possibility of a modification in spleen seeding efficiency was excluded. Thymus cells treated with anti-Thy 1.2 did not abrogate the effect, so the co-operating cell type lies in the minority population of early T cells, thymic macrophages or epithelial cells. Moore (New York) considered the growth and replication of the committed precursor cell, and demonstrated the importance of their genetic origin. NZB mice for example give good colony growth with L-cell-conditioned medium, while giving none with WEHI-3CM, which is perfectly adequate for growth of CBA or NZW CFU-C. On the other hand, hybrids of NZB with either CBA or NZW grow perfectly well in WEHI-3CM. The same patterns held in long-term "Dexter" cultures, where NZB marrow will grow on $W/W^v$ adherent layer but not on a NZB or $Sl/Sl^d$ base. Analysis of $W/W^v$ marrow showed that adherent macrophages were the cells responsible for promoting NZB precursor-cell replication.

A session of 3 papers followed, attempting to understand the mechanisms of CFU-S proliferation control. Lord (Manchester) discussed the nature of production of his CFU-S proliferation inhibitor and stimulator. He showed that while the appropriate producer cells are capable of actively synthesizing their respective factors, they cannot produce them in the presence of the opposing factor. At the same time, the two factors are not mutually destructive. Thus, a scheme was evolved in which the relative proximity to, and concentration of, producer cells could enable the interaction of inhibitor and stimulator to regulate the size of the CFU-S population. Neuwirt (Prague, Czechoslovakia) used hydroxyurea to induce a narrow cohort of CFU-S to enter DNA synthesis. He then demonstrated that plasma collected 2–4 h after the onset of DNA synthesis contained an inhibitor which, if injected shortly after the hydroxyurea, prevented the recruitment of that cohort of CFU-S. These results were thus compatible with the suggestion that inhibitor production is induced by the onset of DNA synthesis in the CFU-S compartment. Blackett (Sutton, England) took, as a starting point, that tissue recovery after a cytotoxic insult is the major point of interest, and went on to describe experiments which showed that cyclophosphamide given 3 days before irradiation enhanced CFU-S recovery (5 days after irradiation the difference in CFU-S levels was 1000-fold). Similar patterns of recovery were obtained with different drugs, and clearly the timing of the interval between drug and irradiation was critical. By correlating the number of CFU-S per femur with the granulocyte levels in the marrow and with time, he was able to construct a hysteresis loop (called by the author a "knot diagram" because one gets tied in knots trying to decipher it) which illustrates the principle of feedback regulation of CFU-S number, and the size of which indicates the delay in onset of the feedback mechanism.

The regulation of stem-cell differentiation presents more serious problems, but was tackled at this meeting by Frindel (Villejuif, France). She found that cytosine arabinoside-damaged marrow released a diffusible substance which caused the erythroid/granulocytic ratio of cells in spleen colonies to be increased from 2 (normal marrow graft) to about 8 (ARA-C-treated marrow graft). Radiation-damaged marrow produced the opposite effect, reducing the E/G ratio from 2 to 1·3. These results were interpreted as the result of a differentiation factor(s), the production of which is thought to be independent of the presence of CFU-S. Furthermore, the differentiation factor cannot distinguish between cycling and non-cycling cells. Problems were raised, however, regarding the meaning of differentiation, and the distinction between differentiation and maturation was discussed.

Jasmin (Villejuif, France) studied the behaviour of precursor cells in mice infected with myeloproliferative sarcoma virus (MPSV). He described the cellular changes in the marrow and spleen during the course of infection, and came to the conclusion that the target cell is very early in the haemopoietic cell lineage—certainly the earliest erythroid precursors were affected—and Jasmin queried whether the developing myelofibrosis indicated a depletion of the most primitive stem cells. It was accepted that the early cells were affected, but the question was whether traversing the cell cycle at a normal rate. This agrees with earlier observations (Wibe, 1980) of cell cycle phase-dependent inactivation of proliferating NHIK 3025 cells which are in late S or $G_2$ during exposure to vincristine. In work with Chinese hamster cells, Olah *et al.* (1978) found that plateau cells were more sensitive to vincristine than exponentially growing cells.

Fig. 2B shows surviving fractions after 24h exposures of NHIK 3025 cells in spheroids to 3 concentrations of vincristine. For cell populations from 5 different depths in the spheroid, single-cell surviving fractions are plotted as a function of the average distance from the spheroid surface. One can see that the surviving fractions are slightly higher for cells in the inner region than for cells in the outer parts of the spheroid. Moreover, survival values of cell populations exposed as spheroids to 16 ng/ml vincristine per ml are very close to the survival values after 4 ng/ml. When spheroids were exposed to 4 ng/ml vincristine or 16 ng/ml in the same experiment (*i.e.* using identical spheroids), single-cell surviving fractions in the different parts of the spheroids after the two drug concentrations were almost identical. This can be seen from Fig. 2B, as survival values indicated by open symbols (circles: 4 ng/ml; triangles: 16 ng/ml) were measured in the same experiment (as also for half-filled or filled symbols).

Several different factors may be partly responsible for the dramatic increase in drug resistance of cells exposed in spheroids over exponentially growing monolayers. Such factors may be different shape of individual cells, different drug uptake, possible reduction in drug supply to the inner spheroid cells due to insufficient penetration of drug through the cell mass, different metabolism in spheroid and monolayer cells, protection of spheroid cells by intercellular communication, drug resistance of hypoxic cells, or different cell kinetic parameters. Hardly any of these factors could be the only reason for the big reduction in sensitivity to vincristine.

Although Sutherland *et al.* (1979) found reduced uptake of Adriamycin in spheroids, they showed explicitly that this was not the only reason for Adriamycin resistance in the inner spheroid cells, which it was suggested was due to different metabolic state of the cells, differences in the microenvironment, or the formation of different drug products.

The relatively uniform sensitivity to vincristine through all parts of the spheroid (Fig. 2B), is interesting in view of the different sensitivity to Adriamycin of inner and outer spheroid cells observed by Sutherland *et al.* (1979). The fact that all survival values in the present study were almost independent of both distance from the spheroid surface and drug concentration, and use of a very long exposure time (24 h), rules out the possibility that resistance to vincristine was entirely due to reduced penetration of vincristine through the cell mass. In work with human glioma spheroids and vinblastine (which is closely related to vincristine) Nedermann *et al.* (1980) showed that most of the drug could be found in the outer 100 $\mu$m of the spheroid after a 15min exposure to this drug.

The fact that plateau-phase cells exposed in monolayer culture were even more resistant to vincristine than were cells exposed in the spheroid (Fig. 2) indicates that resting cells and/or very slowly proliferating cells constitute an extremely resistant subpopulation of cells in the spheroid. This is in accordance with the slight increment in surviving fraction with distance from the spheroid surface (Fig. 2B) as the fraction of resting cells is higher in the core than in the outer parts of the spheroids.

The observed resistance to vincristine fits well with the reported resistance of spheroid cells to lethal damage after highly different treatments as $\gamma$-irradiation, hyperthermia, lymphocyte toxicity, and Adriamycin. It might be that these cases of resistance to treatments are all more or less related to the same protective mechanisms. Generally higher tolerance to